# The Busier the Better: Greater Busyness Is Associated with Better Cognition

**DOI:** 10.3389/fnagi.2016.00098

**Published:** 2016-05-17

**Authors:** Sara B. Festini, Ian M. McDonough, Denise C. Park

**Affiliations:** ^1^Center for Vital Longevity, School of Behavioral and Brain Sciences, University of Texas at DallasDallas, TX, USA; ^2^Department of Psychology, The University of AlabamaTuscaloosa, AL, USA

**Keywords:** cognitive aging, busyness, cognitive engagement, episodic memory, middle age, old age

## Abstract

Sustained engagement in mentally challenging activities has been shown to improve memory in older adults. We hypothesized that a busy schedule would be a proxy for an engaged lifestyle and would facilitate cognition. Here, we examined the relationship between busyness and cognition in adults aged 50–89. Participants (*N* = 330) from the Dallas Lifespan Brain Study (DLBS) completed a cognitive battery and the Martin and Park Environmental Demands Questionnaire (MPED), an assessment of busyness. Results revealed that greater busyness was associated with better processing speed, working memory, episodic memory, reasoning, and crystallized knowledge. Hierarchical regressions also showed that, after controlling for age and education, busyness accounted for significant additional variance in all cognitive constructs—especially episodic memory. Finally, an interaction between age and busyness was not present while predicting cognitive performance, suggesting that busyness was similarly beneficial in adults aged 50–89. Although correlational, these data demonstrate that living a busy lifestyle is associated with better cognition.

## Introduction

Everyday conversation frequently touches upon the busyness of daily schedules. People discuss their packed to-do lists and make inferences about the impact of their busy lifestyle on their health and mental function. Often busyness carries a negative connotation, as people tend to complain about their hectic schedules, yet, little scientific work has been done to empirically investigate the construct of busyness and its associations. To fill this gap, the present study: (1) assesses whether busier people tend to have better or worse cognitive performance; and (2) tests whether this relationship with cognition varies with age.

Busyness has been defined as the subjective evaluation of one’s ongoing activity patterns, including reflections about the quantity of one’s unscheduled time and comparisons to what is expected or standard (see Gershuny, 2005; Levine, 2005). Martin and Park (2003) developed a self-report assessment of day-to-day busyness in the Martin and Park Environmental Demands Questionnaire (MPED). This questionnaire yields two scale scores (i.e., Busyness and Routines), and the Busyness scale asks individuals to reflect upon, for example, how frequently they have too many tasks to complete or too little time in the day. The Busyness measure had high internal consistency and external validity, as it was related to medication adherence, employment status, and household size. We utilize this measure of busyness in the present study and examine its relationship to cognition.

Given the pervasive discussion of busyness in everyday life, it is surprising that few studies have assessed busyness. We note that, unlike engagement, which typically bears a positive connotation, busyness carries a more negative undertone, and, at present, the cognitive associations of a busy lifestyle are empirically unknown. Related literature suggests that busyness either could be beneficial or harmful to cognition. Busyness could be related to increased levels of stress, which can have negative consequences on the brain and cognitive function (i.e., allostatic load, see McEwen, 1998). For instance, stress hormones have been shown to have negative neural impacts, with different brain regions showing more vulnerabilities at different points in the lifespan (for a review see Lupien et al., 2009). Moreover, stress has been shown to narrow attention, impair working memory (i.e., potentially by disrupting encoding and maintenance processes), interfere with knowledge acquisition, and degrade perceptual-motor performance (see Staal, 2004). High stress even increased the risk of death, although this was only true in people who viewed stress as harmful (Keller et al., 2012). Thus, it is possible that individuals who are very busy could have inferior cognitive function relative to their less busy counterparts.

Alternatively, more positively, busyness could be related to increased effortful engagement at work, home, and in leisure activities, which can have advantageous consequences on neural health and cognition. Recently, several studies experimentally manipulated lifestyle engagement levels and found benefits for intense, sustained engagement. In the Synapse Project, productive engagement groups that learned digital photography or quilting showed improvements in episodic memory relative to receptive control groups that did little new learning (Park et al., 2014), and this sustained new learning also resulted in more efficient neural processing (McDonough et al., 2015). An iPad training project similarly found that prolonged engagement in learning to use an iPad resulted in improvements in episodic memory and processing speed (Chan et al., 2014). Moreover, participants in the Experience Corps program improved their memory and executive functioning after prolonged mentoring of elementary school students (Carlson et al., 2008), and participants engaging in the Senior Odyssey curriculum improved their processing speed, inductive reasoning, and divergent thinking after sustained mental engagement (Stine-Morrow et al., 2008). Finally, theater training has also been shown to improve memory and problem solving in older adults (Noice et al., 2004).

In addition to these experimental manipulations of engagement, many correlational studies report benefits of high levels of cognitive, social, and physical activities. Benefits include improved cognition, delayed cognitive decline, increased longevity, and reduced risk of various diseases, including dementias (e.g., see Christensen and Mackinnon, 1993; Glass et al., 1999; Singh-Manoux et al., 2003; Valenzuela and Sachdev, 2007; Wilson et al., 2007; Seeman et al., 2011; Small et al., 2012). Furthermore, greater work complexity (i.e., greater opportunity to perform higher cognitive operations; more independent work) has been associated with better cognition and longevity (see Correa Ribeiro et al., 2013; Then et al., 2013; Andel et al., 2015; Massimo et al., 2015). For instance, higher mental demand at work was related to better cognition at baseline and a slower rate of cognitive decline over 8 years (Then et al., 2015).

Based on these experimental and correlational findings, if busyness serves as a proxy for intense, sustained lifestyle engagement, then we would predict that greater busyness would be associated with better cognition. Moreover, because busyness has been shown to differ between middle-aged adults and older adults (see Martin and Park, 2003), we aimed to examine if the effects of busyness on cognition were consistent across adults aged 50–89. It may be that the greatest effects of busyness will be observed in older adults, who tend to have more deficiencies in cognition compared to their younger counterparts (i.e., Park et al., 2002), and thus, may be more sensitive to the effects of busyness. In line with this hypothesis, some studies of activity levels and work complexity have found larger effects in older adults than middle-aged adults and young adults (e.g., Hultsch et al., 1993; Schooler et al., 1999). On the other hand, busyness could be detrimental to cognition if it heightens stress substantially, as prolonged stress is harmful to the central nervous system (i.e., Lupien et al., 2009). Overall, the goal of the current study is to examine the relationship between busyness and cognition. We interpret our results in the context of other relevant literature on busyness, engagement, and activity levels and also discuss hypothetical mechanisms.

## Materials and Methods

### Participants

A total of 330 participants from the Dallas Lifespan Brain Study (DLBS) spanning ages 50–89 were included in this analysis. The DLBS is a large-scale multi-modal assessment of cognition and brain health, structure, and function in healthy adults. The current sample included participants from a highly-screened, elite cohort, as well as a second cohort with more lenient screening criteria. The second cohort was recruited in order to achieve a broader range in variability in various demographic variables, including health, education, and socioeconomic status, and this cohort has participants spanning ages 50–89. This study was approved by the Institutional Review Board at the University of Texas at Dallas and at University of Texas Southwestern Medical Center. All participants provided informed written consent in accordance with the Declaration of Helsinki. See Table 1 for a summary of the sample.

**Table 1:** **Demographic characteristics of the sample, by decade**.

Age group	*N*	Female (*n*)	Male (*n*)	Education (years)	MMSE	Shipley vocab
50–59	86	52	34	15.69	28.64	33.47
60–69	99	62	37	15.77	28.45	34.49
70–79	90	55	35	15.11	28.13	34.20
80–89	55	32	23	15.87	27.55	34.05

Total	330	201	129	15.59	28.26	34.08

*Note. Mean values are reported for Education, the Mini-Mental State Examination (MMSE), and Shipley vocabulary. There were no significant differences in education or Shipley vocabulary between ages. MMSE performance was significantly worse in the 80s than in the other decades, ps < 0.025. MMSE performance was also worse in 70-year-olds than 50-year-olds, p = 0.032. All of these effects fall in the range of normal aging*.

### Materials

A large neuropsychological battery was administered as part of the DLBS. In the present analyses we assessed five core cognitive constructs—processing speed, working memory, episodic memory, reasoning, and crystallized knowledge. We utilized cognitive tasks that loaded well on these constructs, as well as assessments of age, gender, the highest level of education completed (coded into years of education), and busyness.

#### Busyness

Busyness ratings were obtained from the MPED Questionnaire (Martin and Park, 2003). Sample busyness questions include: How busy are you during an average day? How often do you have too many things to do each day to actually get them all done? How often do you have so many things to do that you go to bed later than your regular bedtime? Each question was answered on a 5-point Likert scale, with higher scores indicating greater busyness. An average busyness score was computed based on the answers to the seven busyness questions. Martin and Park (2003) validated this measure on a sample of 121 participants.

#### Processing Speed

##### Digit comparison

Participants viewed two strings of numbers and determined whether they were the same or different (adapted from Salthouse and Babcock, 1991; Hedden et al., 2002). Number strings were either 3, 6, or 9 digits in length, and the dependent variable was the number of items correctly compared in 45 s.

##### Digit symbol

Participants were given a list with a randomized set of digits. A key at the top of the page displayed nine geometric symbols that corresponded to a digit from 1 to 9. Participants were asked to draw the corresponding symbol below each digit as fast as possible (Wechsler, 1997). The dependent measure was the number of items correctly matched in 90 s.

#### Working Memory

##### Spatial working memory

Participants viewed an array of “boxes” on a computer screen and had to maintain the location of a blue token in working memory for accurate performance. The trials varied in set size from 3 to 8 boxes, and the dependent measure was the additive inverse of the number of errors committed. This task was from the Cambridge Neuropsychological Test Automated Battery (CANTAB; Robbins et al., 1994).

##### Spatial recognition memory

A white square appeared on the screen at five different spatial locations, one location at a time. Participants had to update and maintain these spatial locations in working memory. The dependent measure was the number of correct spatial locations that were identified (CANTAB; Robbins et al., 1994).

##### Delayed match to sample

Participants viewed a complex abstract pattern for several seconds and had to select the same pattern out of a possible four choices either simultaneously with the target pattern, immediately following the target pattern, or after a 12 s delay. The dependent measure was the total number of items that were correctly matched (CANTAB; Robbins et al., 1994).

##### Letter number sequencing

The experimenter read a series of letters and numbers to the participant (e.g., 2-M-7-B). When the experimenter stopped speaking, the participant needed to mentally rearrange the information and to say the numbers in ascending order, followed by the letters in alphabetical order (e.g., 2-7-B-M). The dependent variable was the total number of items correctly answered. This task was from the Wechsler Adult Intelligence Scale (WAIS-III; Wechsler, 1997).

##### Operation span

Participants verified whether a math problem was solved correctly and then read a word that followed the math problem. After 2–5 of these problems had been presented, participants wrote down all of the target words that they remembered in the order that they were presented (Turner and Engle, 1989). The dependent variable was the sum of the words recalled in each set of perfectly recalled trials.

#### Episodic Long-Term Memory

##### Verbal recall memory

Participants read aloud 12 words that were presented one-at-a-time on a computer screen. Immediately after the word list, participants recalled as many words as possible (CANTAB; Robbins et al., 1994).

##### Hopkins verbal learning

The experimenter read a list of 12 words aloud, one word every 1.5 s. The list contained four words in three semantic categories, which were presented in random order. Three different dependent measures were collected: (a) immediate recall, in which participants recalled as many words as possible immediately after hearing them, (b) delayed recall, in which participants recalled as many words as possible after a 20-min delay, and (c) delayed recognition, in which participants listened to the experimenter read a list of 24 words aloud (12 target words, 6 semantically-related foils, and 6 unrelated foils) and judged whether or not the word was originally studied. The delayed recognition test was always given after the delayed recall test (Brandt, 1991).

##### Woodcock-Johnson memory for names

Participants attempted to learn the names of novel, imaginary space aliens in a visual-auditory paired-associate memory test (Woodcock and Johnson, 1989). The task became progressively more difficult as each new alien-name pair was learned. Immediate scores reflect the total number of correct aliens identified as participants were progressively learning more new aliens’ names. There were 72 questions in the immediate recognition test, with 12 to-be-learned alien-name pairs total. Delayed scores reflect memory performance after a 20-min delay. There were 36 questions in the delayed recognition test, with each alien tested three times. This assessment of episodic memory was added after data collection on the DLBS had already begun. Consequently, a smaller subset (*n* = 203) of participants has complete data on the episodic memory construct than on the other cognitive constructs.

#### Reasoning

##### Raven’s progressive matrices

In this reasoning task, participants viewed visual patterns and selected a piece that best completed the given pattern (Raven et al., 1998). There were 24 questions, and the accuracy (percentage correct) of the given problems was used as the dependent measure.

##### ETS letter sets

Out of five alternatives, participants were asked to determine which set of letters did not follow the same pattern as the others (Ekstrom et al., 1976). Participants had 14 min to complete a possible 30 problems. The dependent measure was the number of correct items minus one-fourth of the incorrect items.

##### Stockings of Cambridge

This is a computerized CANTAB version (Robbins et al., 1994) of the Tower of London task. Participants had to follow a set of rules to achieve each goal state. The number of problems solved in the minimum number of moves was the dependent measure.

#### Crystallized Knowledge

##### ETS vocabulary

Out of five alternatives, participants selected the word that most closely matched the meaning of the target word (Ekstrom et al., 1976). They had 8 min to answer 36 problems, and the dependent measure was the number of items correctly answered minus one fourth of the incorrect answers.

##### Shipley vocabulary

Out of four alternatives, participants selected the word that most closely matched the meaning of the target word (Zachary and Shipley, 1986). This was an untimed test with 40 questions. The total number of correct items served as the dependent measure.

### Procedure

As part of the DLBS, participants visited the lab to perform cognitive sessions. The cognitive tasks were spaced over 2 days, in a 2–3 h session each day. Participants also completed a battery of surveys at home on an online system.

## Results

### Busyness and Demographic Characteristics

First, we examined whether busyness varied as a function of age, gender, or education. A bivariate Pearson correlation revealed that busyness decreased with age, *r*_(328)_ = −0.214, *p* < 0.001. That is, older adults tended to report being less busy than middle-aged adults. Moreover, an independent samples *t*-test indicated that women reported being busier than men, *t*_(328)_ = 4.245, *p* < 0.001, *d* = 0.48. Finally, assessment of the relationship between busyness and education revealed that busier people tended to be more highly educated, *r*_(328)_ = 0.128, *p* = 0.020.

### Busyness and Cognition

Next, to investigate the relationship between busyness and cognition, we first created constructs of the five cognitive domains by standardizing performance on each task and averaging the *z*-scores across all tasks for a particular cognitive measure. Construct reliability was high for all measures, as indicated by Cronbach’s alpha: processing speed, *α* = 0.81, working memory, *α* = 0.74, episodic memory, *α* = 0.78, reasoning, *α* = 0.72, and crystallized knowledge, *α* = 0.86.

**Figure 1:**
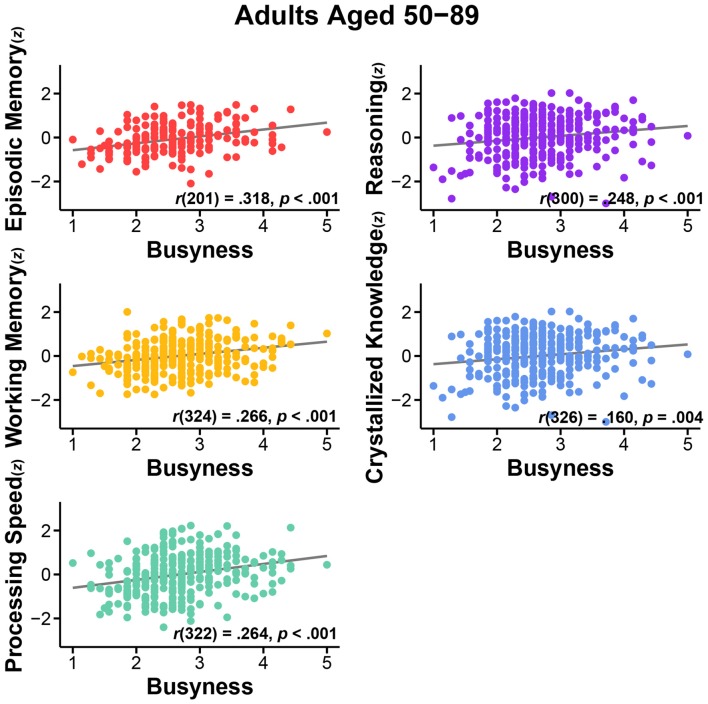
**Relationship between busyness and episodic memory, working memory, processing speed, reasoning, and crystallized knowledge in adults aged 50–89**.

**Figure 2:**
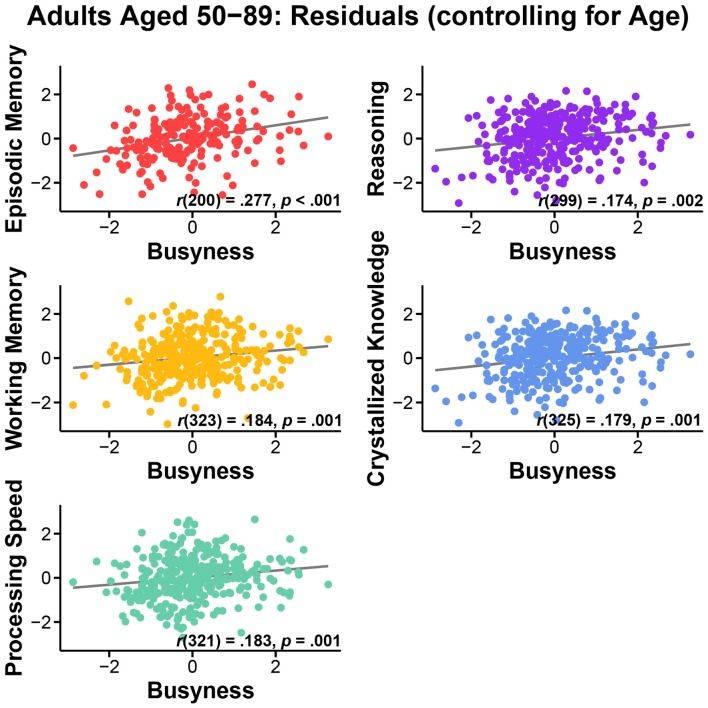
**Relationship between busyness and episodic memory, working memory, processing speed, reasoning, and crystallized knowledge in adults aged 50–89, after controlling for age**.

First, we assessed the nature of the relationship of busyness and cognitive performance. We conducted bivariate correlations between busyness and each of the five cognitive constructs. Results consistently revealed significant positive relationships between busyness and cognition, such that busier people tended to have better cognitive performance. Specifically, greater busyness was associated with faster processing speed, *r*_(322)_ = 0.264, *p* < 0.001, better working memory, *r*_(324)_ = 0.266, *p* < 0.001, better episodic memory, *r*_(201)_ = 0.318, *p* < 0.001, better reasoning, *r*_(300)_ = 0.248, *p* < 0.001, and better crystallized knowledge, *r*_(326)_ = 0.160, *p* = 0.004 (see Figure 1). All relationships remained significant after controlling for age: processing speed, *r*_(321)_ = 0.183, *p* = 0.001, working memory, *r*_(323)_ = 0.184, *p* = 0.001, episodic memory, *r*_(200)_ = 0.277, *p* < 0.001, reasoning, *r*_(299)_ = 0.174, *p* = 0.002, crystallized knowledge, *r*_(325)_ = 0.179, *p* = 0.001 (see Figure 2).

Next, hierarchical linear regressions were run to determine if busyness predicted significant additional variance in cognition that was unexplained by age and education. Across all five analyses, busyness explained significant additional variance in cognition. Notably, busyness had the largest effect on episodic memory, after controlling for age and education, *R^2^* = 0.173, *R^2^ change* = 0.073, *p* < 0.001. Busyness also accounted for significant additional variance in processing speed, *R^2^* = 0.303, *R^2^* *change* = 0.021, *p* = 0.002, working memory, *R^2^* = 0.315, *R^2^ change* = 0.020, *p* = 0.003, reasoning, *R^2^* = 0.259, *R^2^* *change* = 0.018, *p* = 0.007, and crystallized knowledge, *R^2^* = 0.198, *R^2^ change* = 0.016, *p* = 0.011^1^ (see Table 2).

**Table 2:** **Hierarchical regressions with age, education, and busyness as predictors of the five cognitive constructs**.

		Unstandardized		Standardized
Cognitive construct	Predictor	*B*	*Std. Error*	*Beta*	*R^2^*	*R^2^* change	*Sig. F change*
Processing speed	**Age**	−0.043	0.004	−0.489	0.276	0.276	<0.001
	Education	0.025	0.019	0.062	0.282	0.006	0.091
	**Busyness**	0.203	0.066	0.148	0.303	0.021	0.002
Working memory	**Age**	−0.033	0.003	−0.491	0.279	0.279	<0.001
	**Education**	0.033	0.014	0.110	0.296	0.016	0.006
	**Busyness**	0.150	0.050	0.144	0.315	0.020	0.003
Episodic memory	**Age**	−0.015	0.004	−0.237	0.074	0.074	<0.001
	**Education**	0.042	0.022	0.126	0.100	0.025	0.019
	**Busyness**	0.271	0.064	0.275	0.173	0.073	<0.001
Reasoning	**Age**	−0.032	0.004	−0.413	0.202	0.202	<0.001
	**Education**	0.065	0.018	0.180	0.240	0.039	<0.001
	**Busyness**	0.166	0.061	0.140	0.259	0.018	0.007
Crystallized knowledge	Age	0.011	0.005	0.120	0.004	0.004	0.227
	**Education**	0.165	0.020	0.406	0.182	0.177	<0.001
	**Busyness**	0.184	0.072	0.131	0.198	0.016	0.011

*Note. Significant independent predictors are indicated in bold font*.

### Does the Relationship Between Busyness and Cognition Vary with Age?

Finally, a series of hierarchical linear regressions were performed to investigate if age by busyness interactions were present, which would suggest that busyness had a different effect as a function of age. In the regression models, the effects of age and busyness were entered, followed by the interaction term, after controlling for education. Both age and busyness were centered before computing the interaction. The age by busyness interaction was not significant for any cognitive construct: processing speed (*p* = 0.999), working memory (*p* = 0.083), episodic memory (*p* = 0.490), reasoning (*p* = 0.203), crystallized knowledge (*p* = 0.178).^2^ Thus, the relationship between busyness and cognition was similar in adults aged 50–89.

## Discussion

This study was conducted to examine if greater busyness was associated with superior or inferior cognition. Analysis of over 300 people from the DLBS revealed that greater busyness was correlated with better cognition, with the largest effects observed for episodic memory. Furthermore, busyness was similarly influential in adults aged 50–89, indicating that cognitive associations of lifestyle engagement were consistent across this age range. Next, we discuss how our results relate to prior literature, offer several potential mechanisms of the observed effects, and outline future lines of research to consider.

### Greater Busyness is Associated with Superior Cognition

Although busyness is frequently discussed in everyday conversation, little empirical work has examined the cognitive repercussions of a busy lifestyle. Consistent with an engagement framework, the present study revealed that higher levels of busyness were associated with better cognition in adults aged 50–89, with the biggest effects observed for episodic memory. Individuals who reported greater day-to-day busyness tended to have better processing speed, working memory, episodic memory, reasoning, and crystallized knowledge, and these relationships persisted after controlling for age. Moreover, hierarchical regressions demonstrated that after accounting for variations in cognitive ability already explained by age and education, busyness accounted for significant additional variance in all cognitive domains. The most pronounced effects for episodic memory parallel the findings from several experimental engagement protocols (Carlson et al., 2008; Chan et al., 2014; Park et al., 2014), and they are also consistent with many correlational studies that report relationships between participation in cognitive, social, and physical activities and memory (e.g., Hultsch et al., 1999; Buchman et al., 2008; Seeman et al., 2011).

We acknowledge that our analyses are correlational, and while it is notable that busyness and cognition are related, we are unable to determine if living a busy lifestyle improves cognition or if smarter individuals are capable of partaking in more activities, resulting in greater levels of busyness. This is a drawback of all correlational studies, which has been noted in many studies of activity levels (e.g., see Scarmeas and Stern, 2003; Small et al., 2012), yet, the association between busyness and cognition is still worthy of attention, as it offers a rather simple assessment of lifestyle engagement that has implications for cognitive abilities, one that also has high relevance to everyday life.

Our findings are consistent with studies using other measures of engagement. With regard to assessments of activity levels, a myriad of studies have documented significant relationships between activity frequencies and cognitive function (for a review see Fratiglioni et al., 2004). For example, Buchman et al. (2008) report that higher total daily activity predicted better perceptual speed, working memory, episodic memory, visuospatial abilities, and semantic memory. Newson and Kemps (2005) similarly found that greater engagement in general lifestyle activities predicted better incidental recall, among other cognitive processes. Furthermore, Hultsch et al. (1993) report that greater engagement in everyday activities was related to better working memory, episodic memory, verbal fluency, reading comprehension, and vocabulary. And engagement in social activities, has been shown to predict better episodic memory and executive functioning (Seeman et al., 2011), as well as superior cognition in general (Singh-Manoux et al., 2003).

All of these studies relate cognitive, social, and physical activity levels to current levels of cognition, as was done in our present busyness analyses, yet evidence also exists for a relationship between activity frequencies and maintenance of cognition over time. For instance, an engaged lifestyle and high cognitive performance at midlife predicted high cognitive performance in old age (Schaie, 1984). Moreover, decreases in cognitive lifestyle activities have also been shown to predict declines in verbal speed, episodic memory, and semantic memory (Small et al., 2012; see also Hultsch et al., 1999; Newson and Kemps, 2005). Finally, older adults with more lifetime experiences and engagement have been shown to have less cognitive decline over time (see Pushkar Gold et al., 1995; Arbuckle et al., 1998; Schooler and Mulatu, 2001; Zunzunegui et al., 2003; Valenzuela and Sachdev, 2007). Future work should examine whether busyness similarly predicts longitudinal cognitive change. Evidently a relationship exists between activity levels and cognition, and we add to this knowledge by chronicling a similar relationship between busyness and cognition.

However, not all prior studies have found such a pattern. For instance, Wilson et al. (2003) did not find a relationship between lifetime cognitive activities and episodic memory or working memory in older adults. Moreover, Aartsen et al. (2002) report that everyday social, experiential, and developmental activities did not relate to longitudinal change in cognition. Finally, Soubelet and Salthouse (2010) did not find evidence for a relationship between busyness and fluid intelligence, memory, or speed. We are unsure why our findings differ from those reported by Soubelet and Salthouse, considering that they used the same busyness scale and included a large sample with a broad age range. We propose that perhaps our inclusion of a sample with more lenient screening criteria allowed for more variability in both busyness and cognition, which may have contributed to the results.

Finally, although our study found that greater busyness predicted better cognition, especially for episodic memory in the laboratory, this does not suggest that all everyday cognitive activities will exhibit similar benefits. Most notably, busyness may negatively impact prospective memory (i.e., remembering to complete tasks in the future), which was not assessed in the present study. Prior work has shown that busier people tend to have poorer medication adherence (Park et al., 1999; Denhaerynck et al., 2007), and that busier people report more errors of prospective and retrospective memory (Gondo et al., 2010; cf. Cuttler and Graf, 2007, who report that prospective memory was unrelated to busyness and routines). Yet, Neupert et al. (2011) found that, while older adults remembered to take their medications more on days when they were less busy, younger adults remembered to take their medications more when they were busier. Thus, effects of busyness on prospective memory need not follow the pattern observed for episodic memory, and age differences may be present. Furthermore, it is possible that busyness could induce a state of perpetual multi-tasking/dual-tasking. Although busy people may have improved cognition while focusing on a single task, their busy lifestyle may increase distractibility, resulting in worse performance in some situations. This effect of busyness on distractibility remains to be tested. Last, we note that high levels of busyness may limit time for relaxation or self-reflection, both of which can have positive benefits to the self and to cognition (e.g., Grant et al., 2002; Galvin et al., 2006). Nevertheless, busy people may still find opportunity for such outlets (i.e., during exercise or a commute), but additional empirical work is needed to examine this possibility.

### Effect of Busyness was Consistent Across Age

In this study, we also examined whether a significant interaction was present between busyness and age while predicting cognition—that is, whether busyness was differentially related to cognitive performance at different ages. Results indicated no significant interaction for any of our cognitive constructs. Correspondingly, our data suggest that greater busyness was similarly associated with better cognition across adults aged 50–89. This is consistent with Buchman et al. (2008), who report no interaction between daily activity levels, age, gender, or education on cognition, although their study was restricted to older adults, and with Seeman et al. (2011) who found that social engagement was similarly beneficial in a sample of both middle-aged and older adults. Thus, our findings have implications for the benefits of living an active, busy lifestyle throughout middle and late adulthood.

### Potential Mechanisms of Improved Cognition with Busyness

We acknowledge that the correlational nature of the present study does not allow us to definitively address mechanisms underlying the relationship between busyness and cognition. It is indeed possible that individuals with better cognition seek out or are able to sustain more busy lifestyles, rather than that high levels of busyness facilitate cognitive function. We also recognize that an additional, unexplored factor could be contributing to the observed association. Nevertheless, given evidence from prior research on learning and engagement training, we consider several possible mechanisms of how busyness could promote cognition. These potential mechanisms can be tested with future work.

First, prior studies have shown that new learning promotes the retention of new neurons in the hippocampus (see Churchill et al., 2002; Shors, 2014). Busyness may similarly increase the opportunity for new learning, as a busy person is likely to be exposed to more information and more types of situations on a daily basis. According to the Gallup Poll, people who reported experiencing high levels of stress yesterday were more likely to also say that they learned something interesting that day (see McGonigal, 2015). This potential abundance of new learning in busy people may contribute to the maintenance of new hippocampal neurons, which may assist episodic memory. This hypothesis is consistent with the sustained engagement findings in the Synapse project (Park et al., 2014), as differentially greater improvements in episodic memory were observed in individuals who partook in digital photography or quilting training as compared to individuals who partook in frequent social interaction, where little new learning was required (see also Chan et al., 2014).

Second, busyness could promote the development of *neural scaffolding* and consequently facilitate cognition (Park and Reuter-Lorenz, 2009; Reuter-Lorenz and Park, 2014). Park et al. (2007) propose that the creation or implementation of neural pathways that did not exist previously may be a mechanism of engagement interventions, and a similar effect is possible for engagement due to busyness.

Third, in a similar vein, busyness may promote the development of *cognitive reserve* and *brain reserve* (Scarmeas and Stern, 2003; Stern, 2009; Tucker and Stern, 2011). Living a busy lifestyle could instill the use of more efficient cognitive processing (cf. McDonough et al., 2015) by fostering better cognitive strategies (i.e., cognitive reserve) or expanding processing resource (i.e., neural reserve) to deal with an increased cognitive load, which may assist with overall cognition.

Fourth, in accordance with the *environmental complexity hypothesis* (Schooler, 1984), people who live busy lifestyles may be more likely to encounter more diverse stimuli, may be required to make more complex decisions, and may encounter and have to solve ill-defined problems, which is theorized to promote better cognition.

Finally, busyness may encourage the reliance on memory strategies and aids that may assist performance. In a prospective memory experiment, Uttl and Kibreab (2011) found that people who reported high levels of activities and events (i.e., which could relate to busyness) used memory strategies and aids more frequently. Any combination of these potential mechanisms could contribute to the effects we observed between busyness and cognition, and additional research is needed to test specific mechanisms experimentally.

### Conclusions

We investigated whether busyness was beneficial or detrimental to cognition in adults aged 50–89. Importantly, we document that busier people tend to have better cognition, especially episodic memory. Although correlational in nature, these results are in line with an engagement framework, and they have implications for the usefulness of engagement training programs. Additional experimental work should be conducted to determine if manipulations of busyness influence cognition in a similar manner. Overall, our findings offer encouragement to maintain active, busy lifestyles throughout middle and late adulthood.

## Author Contributions

SBF designed the study, analyzed the data, interpreted the results, and wrote the manuscript. IMMcD offered suggestions for analyses, helped interpret the results, and also critically edited the manuscript. DCP designed the study, helped interpret the results, and critically edited the manuscript. All authors approve the final version of the manuscript and agree to be accountable for the content of the work.

## Funding

This study was supported by NIH Grant 5R37AG-006265-29 awarded to DCP. SBF is supported by the Aging Mind Foundation.

## Conflict of Interest Statement

The authors declare that the research was conducted in the absence of any commercial or financial relationships that could be construed as a potential conflict of interest.

## Footnotes

^1^All effects remained significant when additionally controlling for cohort. Similarly, all effects remained significant when additionally controlling for gender, with the exception that the effect of busyness on processing speed became marginal, *p* = 0.058. When additionally controlling for both cohort and gender, the effect of busyness on processing speed became non-significant, *p* = 0.145.

^2^All interactions remained non-significant after additionally controlling for both cohort and gender.
