# Corrigendum: The Busier the Better: Greater Busyness Is Associated with Better Cognition

**DOI:** 10.3389/fnagi.2016.00148

**Published:** 2016-06-20

**Authors:** Sara B. Festini, Ian M. McDonough, Denise C. Park

**Affiliations:** ^1^Center for Vital Longevity, School of Behavioral and Brain Sciences, University of Texas at DallasDallas, TX, USA; ^2^Department of Psychology, The University of AlabamaTuscaloosa, AL, USA

**Keywords:** cognitive aging, busyness, cognitive engagement, episodic memory, middle age, old age

Reason for Corrigendum:

In the originally published article, the scatterplots for Reasoning were incorrect in Figures 1, 2, although the reported statistics were accurate. The corrected Reasoning scatterplots appear in Figures 1, 2 below. This error does not change the scientific conclusions of the article in any way. The authors regret the mistake.

**Figure 1:**
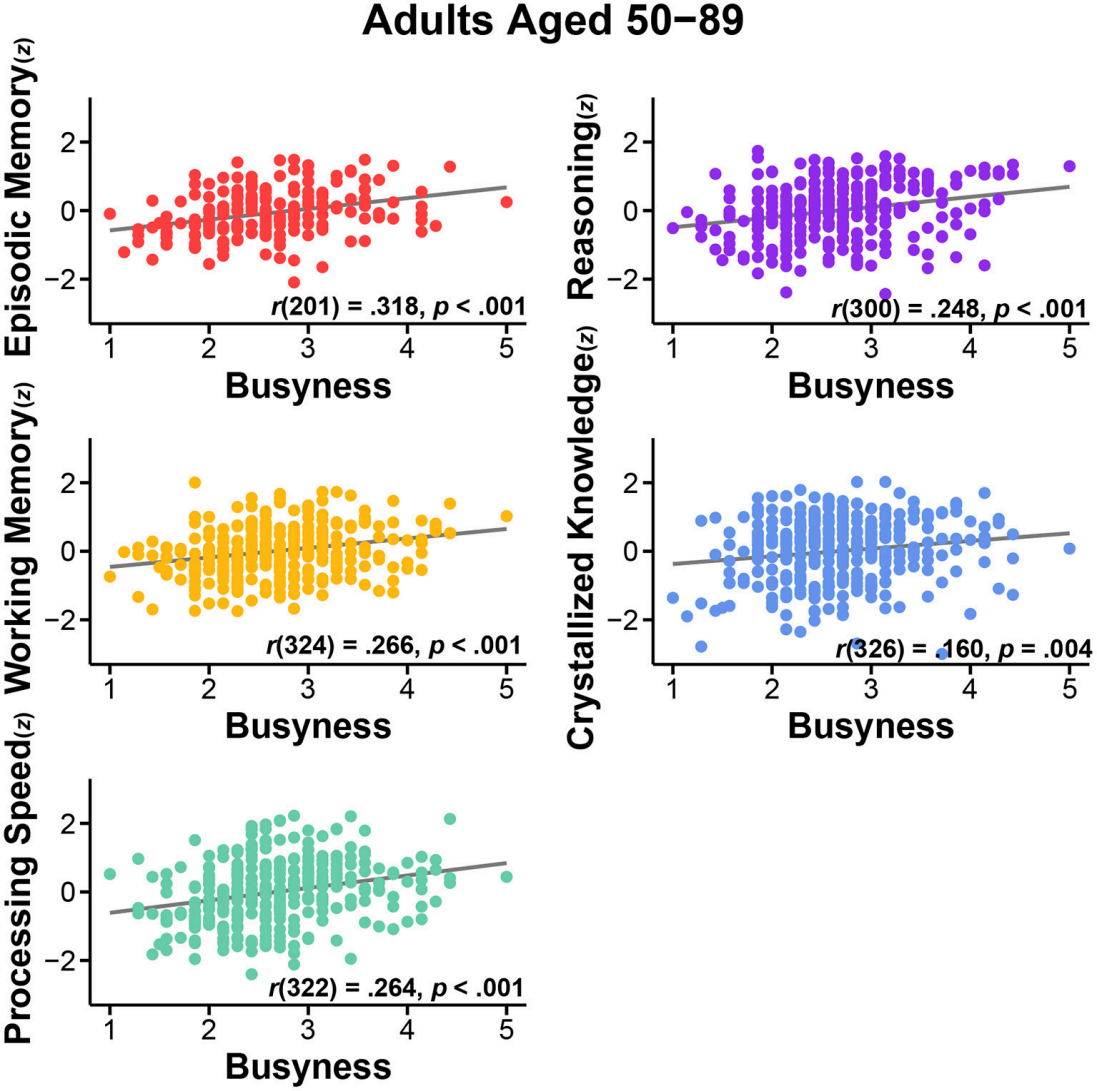
**Relationship between busyness and episodic memory, working memory, processing speed, reasoning, and crystallized knowledge in adults aged 50–89**.

**Figure 2:**
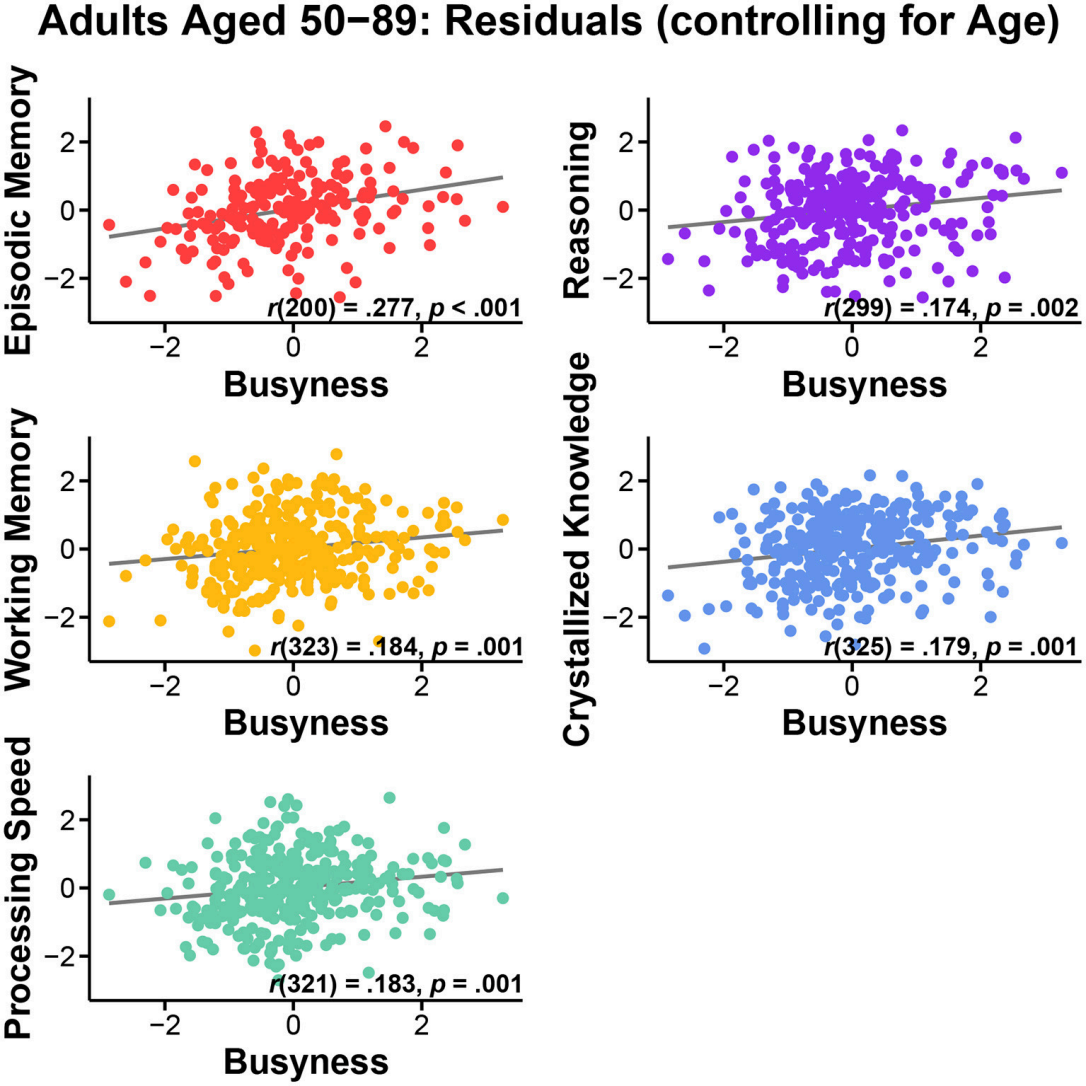
**Relationship between busyness and episodic memory, working memory, processing speed, reasoning, and crystallized knowledge in adults aged 50–89, after controlling for age**.

## Author contributions

SBF designed the study, analyzed the data, interpreted the results, and wrote the manuscript. IMMcD offered suggestions for analyses, helped interpret the results, and also critically edited the manuscript. DCP designed the study, helped interpret the results, and critically edited the manuscript. All authors approve the final version of the manuscript and agree to be accountable for the content of the work.

## Funding

This study was supported by NIH Grant 5R37AG-006265-29 awarded to DCP. SBF is supported by the Aging Mind Foundation.

### Conflict of interest statement

The authors declare that the research was conducted in the absence of any commercial or financial relationships that could be construed as a potential conflict of interest.

